# Diagnosis and Treatment of von Willebrand Disease and Rare Bleeding Disorders

**DOI:** 10.3390/jcm6040045

**Published:** 2017-04-10

**Authors:** Giancarlo Castaman, Silvia Linari

**Affiliations:** Center for Bleeding Disorders and Coagulation, Department of Oncology, Careggi University Hospital, Largo Brambilla 3, 50134 Florence, Italy; linaris@aou-careggi.toscana.it

**Keywords:** inherited disorder, von Willebrand factor, clotting factor deficiency, bleeding, desmopressin, replacement therapy, on-demand, prophylaxis

## Abstract

Along with haemophilia A and B, von Willebrand disease (VWD) and rare bleeding disorders (RBDs) cover all inherited bleeding disorders of coagulation. Bleeding tendency, which can range from extremely severe to mild, is the common symptom. VWD, due to a deficiency and/or abnormality of von Willebrand factor (VWF), represents the most frequent bleeding disorder, mostly inherited as an autosomal dominant trait. The diagnosis may be difficult, based on a bleeding history and different diagnostic assays, which evaluate the pleiotropic functions of VWF. Different treatment options are available for optimal management of bleeding and their prevention, and long-term outcomes are generally good. RBDs are autosomal recessive disorders caused by a deficiency of any other clotting factor, apart from factor XII, and cover roughly 5% of all bleeding disorders. The prevalence of the severe forms can range from 1 case in 500,000 up to 1 in 2–3 million, according to the defect. Diagnosis is based on bleeding history, coagulation screening tests and specific factor assays. A crucial problem in RBDs diagnosis is represented by the non-linear relationship between clinical bleeding severity and residual clotting levels; genetic diagnosis may help in understanding the phenotype. Replacement therapies are differently available for patients with RBDs, allowing the successful treatment of the vast majority of bleeding symptoms.

## 1. Introduction

Inherited bleeding disorders are a heterogeneous group of coagulation disorders characterized by a wide range of frequency and severity of bleeding symptoms, variable inheritance as X-linked disorders (hemophilia) or autosomal in the remainders, and different treatment options. Haemophilia A and B with von Willebrand disease (VWD) represent 95% to 97% of all these diseases, while the remaining part is covered by the so-called rare bleeding disorders (RBDs) [1]. The RBDs are caused by a deficiency of fibrinogen, factor (F) II, FV, FVII, FX, FXI, FXIII or combined FV + FVIII and vitamin K-dependent coagulation factors deficiency (VKCFD).

## 2. von Willebrand disease

VWD represents the most frequent autosomally inherited bleeding disorder, with a prevalence up to 1% on the basis of epidemiological studies [2,3], but most likely lower and around 0.01% based on clinical significance of symptomatic patients [4]. VWD is caused by a deficiency and/or abnormality of von Willebrand factor (VWF), a large multimeric adhesive plasma glycoprotein, that plays an essential role in both primary and secondary hemostasis [5].

### 2.1. Biosynthesis and Physiological Role of von Willebrand Factor

Circulating VWF is composed of a series of dimers of mature constitutive subunits, with a molecular weight ranging from 500 to 20,000 kDa. VWF is encoded for by a gene located on the short arm of chromosome 12 (12p13.2), including about 178 kb with 52 exons [6]. The primary product is a 2813 amino acid protein with a signal peptide of 22 amino acids (prepeptide), a propeptide of 741 amino acids (propeptide), and a mature VWF subunit of 2050 amino acids. Four repeated molecular domains (D1, D2, D’, D3, A1, A2, A3, D4, B, C1, C2) of complementary DNA (cDNA) are responsible for the different binding of the molecule. After cleavage of the prepeptide, the VWF subunits dimerize in the endoplasmic reticulum through intermolecular disulfide bridge at the C-terminal region. Subsequently, the acid pH and high calcium level induce the formation of VWF multimers through disulfide bridges between D3 domains in the Golgi. The propeptide (VWFpp) is cleaved but still remains non-covalently bound to the growing VWF multimer, that previously organizes itself into a right-handed helix and then in a tubular conformation for storage. VWFpp is required for proper intracellular processing of VWF since it participates in the VWF multimerization process and is essential in the regulated storage of VWF [7]. Propeptide (VWFpp) is secreted in a 1:1 ratio to VWF, with a very short half-life (around 2 h compared to approximately 12 h for VWF) [7]. It can be measured in plasma and an increased VWFpp/von Willebrand factor antigen (VWF:Ag) ratio identifies patients with shortened VWF survival after desmopressin (see below).

The biosynthesis of VWF is limited to megakaryocytes and endothelial cells, where the molecule is stored in α-granules or specific organelles called Weibel–Palade bodies [8,9]. Thus, VWF is secreted in plasma and in subendothelial space by a constitutive and a regulated pathway, and proteolysed by a disintegrin and metalloproteinase with a thrombospondin type 1 motif, member 13 (ADAMTS-13), producing an array of multimers ranging from the simple dimer to very large multimers [10]. In vessels with low shear stress VWF circulates in a globular, inactive form. With vascular injury and exposure of subendothelial collagen VWF uncoils in adhesive filamentous forms becoming more accessible to ADAMTS-13 cleavage.

The primary role of VWF is to promote platelet-subendothelium adhesion, platelet-to-platelet interaction and aggregation, promoting further clotting. Adhesion is mediated by the interaction between the A1 domain of VWF and platelet glycoprotein Ib (GpIb) and aggregation of platelets by that with the other receptor on platelets GpIIb/IIIa, which after activation binds to VWF and fibrinogen, with further recruitment of platelets into a stable plug [11].

**Figure 1:**
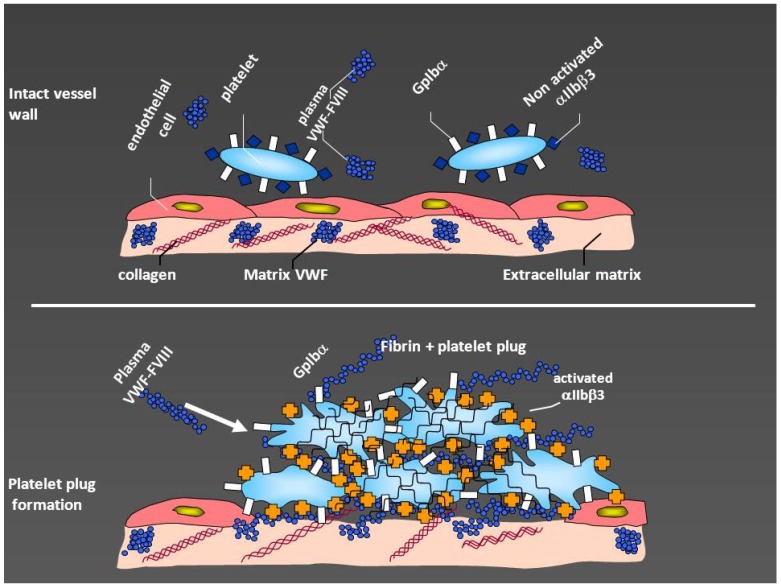
The role of von Willebrand factor (VWF) in hemostasis. After vessel injury, subendothelium becomes exposed with collagen and VWF contained in the subendothelial matrix. The interaction of von Willebrand factor with glycoprotein Ibα (GpIbα), naturally exposed on the platelet surface, creates initial adhesion of platelets to the subendothelium and platelet activation. This makes the platelets to expose glycoprotein IIb/IIIa (αIIbβ3) which binds fibrinogen and VWF thus, increasing platelet plug. Factor VIII is bound to VWF and is naturally conveyed to the site of lesion, thus allowing also the generation of adequate amounts of fibrin (modified by courtesy of AB Federici).

The other critical function of VWF is to serve as the carrier protein for coagulation FVIII, protecting it from proteolytic degradation and prolonging its half-life in plasma and correctly localizing it at the site of damage [12,13]. Figure 1 summarizes these physiological roles.

Considering the haemostatic pleiotropic functions of VWF, its quantitative and/or qualitative abnormalities can be associated with a substantial risk of bleeding, whose severity is usually proportional to the degree of the primary deficiency of VWF and to that of secondary deficiency of FVIII.

### 2.2. Classification of von Willebrand Disease

VWD is a heterogeneous disorder and is usually classified into three main types according to quantitative (Types 1 and 3) or qualitative (Types 2A, 2B, 2M, 2N) abnormalities (Table 1) [14,15]. Type 1 is easily distinguished from type 3 by its milder deficiency, that is usually in the range of 20%–40%, the autosomal dominant inheritance transmission with the majority of mutations as missense and the presence of milder bleeding manifestations. Several guidelines suggest that levels below 30% are diagnostic for VWD, while between 30% and 50% are considered as “low VWF” with a mild risk of bleeding [16]. Type 3 VWD is characterized by total absence of VWF in plasma and platelets; in 80% of type 3 patients, the genetic defects are null alleles in the *VWF* gene [17], and most of these patients are homozygous or compound heterozygous.

Among type 2 variants, four subtypes have been described on the basis of different pathophysiological mechanisms (Table 1). The vast majority of type 2 VWD are autosomal dominant disorders with high penetrance and expressivity caused by missense mutations, while types 3 and 2N are usually inherited in an autosomal recessive manner.

### 2.3. Clinical Manifestations

Clinical expression of VWD is usually mild in type 1, with progressive increasing severity in type 2 and 3. The severity of bleeding tendency is usually proportional to the degree of the primary deficiency of VWF and to that of secondary deficiency of FVIII.

Mucocutaneous bleeding (epistaxis especially during childhood, menorrhagia, easy bruising) are typical, prominent manifestations of the disease and may affect the quality of life. Since FVIII is usually only mildly reduced, manifestations of a severe coagulation defect (haemarthrosis, deep muscle hematomas) are rare except in type 3 VWD, where the severity of bleeding may be similar to that of moderate hemophilia. Gastrointestinal bleeding may be particularly frequent and difficult to manage, especially in patients lacking high molecular weight (HMW) multimers in plasma [18]. Bleeding after dental extraction is the most frequent post-operative bleeding manifestation, whereas bleeding after surgery may occur in more severely affected type 1 and 3 VWD patients. Bleeding after delivery is rarely observed in type 1 since FVIII/VWF levels tend to correct at the end of pregnancy in mild type 1 cases, whereas types 2A, 2B and 3 females usually require replacement therapy post-partum to prevent immediate or delayed bleeding.

### 2.4. Diagnosis of von Willebrand Disease: Bleeding History and Laboratory Evaluation

The diagnosis of VWD, and in particular of type 1, may be difficult due to clinical heterogeneity and difficulties in standardizing diagnostic tests. VWF levels are also significantly influenced by physiological (age, exercise and pregnancy) or pathological (inflammation and cancer) variables. Furthermore, blood group plays a major role in plasma VWF levels, in fact they are 25%–35% lower in type-0 individuals than in non-0 [19,20].

The hallmark of VWD may be considered a history of mucocutaneous bleeding symptoms, that is a necessary requirement to initiate a laboratory assessment. Data on large cohorts of VWD patients have demonstrated that a positive bleeding history is highly specific (>95%) but less sensitive (50%–60%) for the presence of the disease. A bleeding history can be suggestive for VWD when at least three different haemorrhagic symptoms are reported or the bleeding score is greater than 3 in males or greater than 5 in females [21]. The bleeding score (BS) is a summative index accounting for both the number and the severity of bleeding that is generated by summing the severity of all reported bleeding symptoms and graded according to an arbitrary scale [22]. A novel questionnaire has been recently endorsed by the International Society on Thrombosis and Haemostasis (ISTH) to assess bleeding symptoms for the diagnosis of bleeding disorders [23].

The diagnosis of VWD is based on different diagnostic assays, which evaluate the pleiotropic function of VWF and are summarized in Table 2, according the VWF Scientific and Standardization Subcommitte of the ISTH [24]. Whatever, the basic diagnostic tests are the measurements of VWF:Ag, the level of VWF-dependent platelet adhesion, historically evaluated using the VWF-ristocetin cofactor activity assay (VWF:RCo) and the coagulant activity of FVIII (FVIII:C). The propeptide assay, still mainly used for experimental purposes, may help to identify patients with a possible increased VWF clearance when basal VWFpp/VWF:Ag ratio is >2.5–3.

In VWD patients, the platelet count is usually normal, only in type 2B a mild to moderate thrombocytopenia can be found. The bleeding time (BT) is usually increased, the prothrombin time (PT) is normal, whereas the activated partial thromboplastin time (APTT) may be prolonged, depending on the plasma FVIII levels. Type 1 VWD is characterized by a mild to moderately severe reduction in plasma of both VWF:Ag and VWF:RCo.; VWF is functionally normal, as is the pattern of plasma VWF multimers, and plasma levels of FVIII are usually reduced in proportion to VWF. Type 2A is characterized by a low VWF:RCo/VWF:Ag ratio (<0.6) with lack of large and intermediate size of VWF multimers and impaired ristocetin-induced platelet aggregation (RIPA). The laboratory hallmark of the most typical and frequent forms of type 2B is the heightened RIPA with mild to moderate thrombocytopenia, mildly reduced to normal FVIII and VWF:Ag, reduced VWF:RCo and absence of the HMW multimers in plasma. In type 2M VWD the VWF multimer distribution is normal, but platelet-dependent VWF activities (VWF:RCo and VWF:CB) are reduced. Type 2N is characterized by recessive inheritance and slightly reduced VWF:Ag and VWF:RCo, with an intact multimeric pattern. Low plasma levels of FVIII:C (tipically 5–40 U/dL) result from the increased clearance of this moiety, which cannot bind to VWF as a consequence of its qualitative abnormality. Type 3 VWD is characterized by undetectable levels of VWF (usually <3 U/dL) and very low levels of FVIII:C (tipically 1–5 U/dL).

In practice the laboratory diagnosis of VWD is based on the presence of reduced VWF:RCo (or VWF:CB) (<40 U/dL), with a further characterization of VWD type based on the assessment of VWF:Ag, FVIII:C and multimer pattern. VWF levels below 30 U/dL have been shown to be strongly associated with a significant clinical severity [20] and the presence of the mutations in *VWF* gene [25].

### 2.5. Management of Patients with von Willebrand Disease

Effective treatment in VWD requires the correction of the dual haemostatic defect in case of bleeding or before an intervention. Current therapeutic strategies involve either elevation of the plasma concentrations through the release of endogenous VWF from endothelial cells with desmopressin (DDAVP) or replacement therapy of VWF with human plasma-derived (pd) low-purity FVIII-VWF concentrates or a high-purity VWF product. Other treatments can be considered additional to these two modalities.

DDAVP (1-deamino-8-d-arginin-vasopressin), a synthetic analog of the antidiuretic hormone vasopressin, V2 agonist [26], increases VWF and FVIII in plasma without significant side effects. Other important advantages of the drug are its safety from the risk of transmitting blood-borne viruses and its low cost. DDAVP can be administered intravenously (0.3 mcg/kg diluted in 50–100 mL saline and infused over 30 min), subcutaneously (0.3 mcg/kg), or intranasally (fixed doses of 300 mcg in adults and 150 mcg in children). This treatment increases circulating FVIII/VWF three to five times above basal levels within 30–60 min; high plasma FVIII/VWF concentrations last for 6–8 h. DDAVP dose can be repeated after 12 to 24 h, depending on the individual response, which is influenced by various factor, including genotype and phenotype [27]. Therefore, a test-dose infusion at the time of diagnosis is recommended to establish the individual response pattern and its duration. Even among initially DDAVP-responder patients, a short half-life of VWF could be observed. These patients are usually identified on the basis of increased VWFpp/VWF:Ag at baseline. During repeated treatments, a progressive reduced response to DDAVP can be observed. This tachyphylaxis is due to depletion of VWF/FVIII from the storages. Side effects, attributable to the vasomotor effect of the molecule, may include mild tachycardia, flushing, and headache. Rare side effects, due to the antidiuretic properties of DDAVP, are hyponatremia, especially in children below the age of 2, and volume overload, both preventable by limiting fluid intake for 24 h after the administration of DDAVP [26]. No cardiovascular episodes have been described in VWD patients treated with DDAVP; however, the drug should be used with caution in elderly subjects with atherosclerotic disease, because a few cases of stroke and myocardial infarction occurred in haemophiliacs and uremic patients [28].

DDAVP is usually effective in patients type 1 VWD and baseline VWF and FVIII levels higher than 10 IU/dL [29]. In type 2B, DDAVP is generally contraindicated because of the transient appearance or aggravation of thrombocytopenia leading to an increased risk of bleeding [30]. Patients with type 3 VWD are unresponsive to DDAVP.

For those VWD patients in whom DDAVP is either ineffective or contraindicated, replacement therapy with pd-VWF/FVIII concentrates is the treatment of choice. Current effective intermediate and high-purity VWF-FVIII concentrates are several and all plasma-derived [31], with a variable content of VWF and FVIII, as well as a heterogeneous multimer pattern (Table 3). Due to the different production techniques, the retention or loss of HMW VWF multimers, responsible for the most adhesive or functional forms of VWF, may be significantly different with theoretically better hemostatic efficacy for those with better multimer profile [32]. However, there is no clinical evidence that the various pd-VWF/FVIII products differ regarding hemostatic efficacy and in terms of pharmacokinetics [33]. Different recommendations or national guidelines for VWD treatment are available, and useful indications for replacement therapy in spontaneous bleedings, minor and major surgeries, delivery and puerperium, development of alloantibodies [34] are summarized in Table 4 [24,35]. For patients with milder deficiencies and not candidate to DDAVP, treatment is usually less stringent and prolonged and lower doses are required according to the actual basal levels. The required dose should however always be monitored by assaying FVIII and VWF as needed.

Repeated infusions of pd-VWF/FVIII for severe bleedings or major surgery may result in a significant accumulation of FVIII, that is exogenously infused and endogenously synthesized and stabilized by the infused VWF [36], with possible occurrence of deep vein thrombosis or cardiovascular problems [37,38]. To avoid this adverse event, a daily monitoring of FVIII:C plasma level may be recommended to maintain levels below 150 IU/dL [39]. Alternatively, the high-purity VWF concentrate with low amounts of FVIII can be used, with co-administration of a priming dose of FVIII, when prompt hemostasis is required in patients with baseline FVIII:C levels lower than 30 IU/dL, since endogenous FVIII needs 6–8 h to reach its peak after infusion [40]. Therefore, when pd-VWF/FVIII are used in patients with VWD, personalized treatments are often necessary to obtain hemostatic efficacy avoiding adverse effects. A recombinant VWF concentrate, characterized by the absence of the theoretical risk of pathogen transmission and by homogeneity of content in VWF and HMW multimers [41], has recently completed successful clinical trials [42,43]. However, no specific indications on its clinical use are so far available.

In contrast to patients with haemophilia, regular replacement therapy to prevent bleeding is not commonly used in those with VWD, even if its benefit has been described in several case series [44,45]. In patients with severe forms of VWD, when plasma FVIII level is <5 U/dL, suffering from recurrent joint bleeding or gastrointestinal bleeding, which may also affect patients with type 2 devoid of HMW multimers [18] due to a frequently associated enteric angiodysplasia [46], a secondary long-term prophylaxis may represent an efficacious treatment. Even children with frequent and severe epistaxis, responsible for anemia could be candidates [47,48]. Usually doses of at least 20–40 U/kg twice weekly are used and, to avoid accumulation of FVIII, concentrates rich of VWF (or recombinant VWF) with low or no FVIII content could be considered.

Additional important treatment of mucocutaneous bleeding is represented by the fibrinolysis inhibitor tranexamic acid [49] at dosage of 15–25 mg/kg every 8 h for 3–6 days by oral or intravenous administration. In addition to systemic treatment for bleeding or surgery, the agent can be used as mouthwash for oral bleeding or dental procedures.

To control gynaecologic bleeding, hormonal therapy is often helpful [50]. Treatment should be initiated with oral contraceptives containing both progestin and estrogen. In those patients who do not have a significant response, a levonorgestrel-releasing intrauterine device can be used [51].

## 3. Rare Bleeding Disorders

RBDs cover 3%–5% of all inherited coagulation deficiencies and are characterized by an extreme variability of bleeding manifestations, ranging from mild to severe even in patients affected by the same disorder. These disorders are usually transmitted as autosomal recessive conditions, but some cases of FXI deficiency, hypofibrinogenemia and dysfibrinogenemia can be autosomal dominant [52]. The prevalence of the severe forms range from 1 patient in 500,000 for FVII up to 1 in 2–3 million for FII and FXIII deficiencies (Table 5). RBDs are described in most populations, with a higher frequency in those where consanguineous marriages are common [52]. According to epidemiologic data collected from World Federation of Haemophilia (WFH) and European Network of the Rare Bleeding Disorders (EN-RBD), the prevalence of each deficiency in total affected population is the following: FVII 39%, FXI 26%, fibrinogen, FV and FX 8%–9%, FXIII 6%, combined FV + FVIII 3% and FII 1% [52,53].

Hemorrhagic manifestations are heterogeneous, anyway mucosal bleeding and bleeding at the time of invasive procedure or surgery are common to all RBDs, as, in affected women, menorrhagia, spontaneous abortion and bleeding at delivery. Gastrointestinal bleeding, hematomas and haemarthrosis are more frequent in patients with FX deficiency. Serious manifestations, as umbilical cord bleeding and cerebral hemorrhages, occur more frequently in patients with severe defect of fibrinogen, FVII or FXIII (Table 5) [52,53]. It should be borne in mind that FXII deficiency, even in its severe form, is not associated with a bleeding tendency.

### 3.1. Diagnosis of Rare Bleeding Disorders

As in VWD, also in RBDs a positive bleeding history is often the key to suspect diagnosis. However, the evaluation of bleeding symptoms and their severity may represent a challenge, because of their subjective interpretation by patients at least in mild cases in an attempt to standardize bleeding histories, different bleeding score system (BSSs) or bleeding assessment tools (BATs) have been developed, with inconclusive results. Recently, a new promising BAT has been proposed in this setting, but further evaluation of its potential role is needed [54].

Another crucial problem in RBD diagnosis is represented by the non-linear relationship between clinical bleeding severity and residual clotting levels. A strong association can be found in fibrinogen, combined FV + FVIII, FX and FXIII deficiencies, while a weak association for FV and FVII defects. Residual FXI plasma level does not predict clinical bleeding severity [55]. Furthermore, the minimum level to ensure absence of bleeding is different for each RBDs.

Laboratory diagnosis usually starts with the assessment of the coagulation screening tests APTT and PT. A prolonged APTT with normal PT suggests FXI deficiency after exclusion of FVIII, FIX and FXII defects. The reverse pattern is usually due to a FVII deficiency, while the prolongation of APTT and PT together suggests the diagnosis of combined FV + FVIII, FX, FV, FII or fibrinogen deficiencies. To evaluate fibrinogen deficiency thrombin time (TT) is also important. When screening coagulation tests are abnormal, mixing analysis (50:50) must be done to exclude the presence of an inhibitor. Therefore, specific factor assays are performed to identify the deficiency. The diagnosis of FXIII defect requires specific tests, because all screening clotting assays are normal. Increased clot solubility in 5 M urea or 1% monochloroacetic acid may occur when FXIII activity is below 3%–5%. FXIII activity should be evaluated with ammonia release during the transglutaminase reaction or incorporation of radioactive amines in to protein [56]. When FXIII activity is decreased, an immunological FXIII antigen assays is mandatory to establish the deficiency subtype (FXIII-A or FXIII-B) for appropriate diagnosis and treatment (Table 5) [57].

Molecular diagnosis allows to identify the pathogenic mutation in genes that encode corresponding clotting factors. Combined FV and FVIII deficiency is instead due to mutations in genes encoding proteins responsible for intracellular transport of the two coagulation factors (MCFD2 and LMAN1, respectively), while that of VKCFD to mutations in genes encoding necessary enzymes for post-translational change and vitamin K metabolism, namely Gamma-Glutamyl Carboxylase (GGCX) and Vitamin K epoxide reductase (VKOR) [53]. Inheritance pattern is autosomal recessive, except for some cases of hypofibrinogenemia, dysfibrinogenemia and FXI. All identified mutations are collected in the ISTH mutations database [58], which shows that missense mutations cover 50%–80% of cases, insertion/deletion mutations represent 20%–30%, splicing and non-sense mutations comprise the remaining 5%–15%.

### 3.2. Treatment

The availability of specific clotting factor concentrates, except for FII and FV deficiencies, has greatly improved the quality of life of patients affected by RBDs (Table 6). Replacement treatment, using virally inactivated plasma-derived or more rarely recombinant specific factor concentrates, allowed the management of bleeding, the effective prevention of hemorrhages in surgical care and the adoption of medium and long-term prophylaxis in selected cases. When specific clotting factor is not available, treatment may require repeated infusions of fresh frozen plasma (FFP), preferably virus-inactivated, at dosage of 15–20 mL/kg, with potential fluid overload. Alternatively, cryoprecipitate or PCC according to the type of defect could be also used. Dosage and treatment frequency depend on the required minimal haemostatic level of each coagulation factor, its plasma half-life and the type of bleeding treated or to be prevented. The rarity of different RBDs requires that patients are followed in specialized centers, able to provide multidisciplinary assistance for the specific defect and its complications.

### 3.3. Fibrinogen Deficiency

Fibrinogen is a complex adhesive glycoprotein involved in the final step of coagulation cascade, converted into the insoluble fibrin clot by the action of thrombin and FXIII. It also has important interactions with other adhesion molecules, platelets, endothelial cells and cells involved in the inflammatory response. Fibrinogen is produced by liver from 3 homologous polypeptide chains Aα, Bβ and γ, that are assembled to form a hexamer. Each chain is encoded by a separate gene, *FGA*, *FGB*, and *FGG*, which are clustered in a 50-kb region on chromosome 4q32.1. Inherited fibrinogen disorders are classified according to complete or partial quantitative deficiency (afibrinogenemia and hypofibrinogenemia, respectively) or by the presence of an abnormal circulating molecule without or with an associated true quantitative deficiency (dysfibrinogenemia or hypodysfibrinogenemia) [59]. Hypofibrinogenemia (fibrinogen >50–100 mg/dL) represents the heterozygous state for afibrinogenemia.

Patients with hypofibrinogenemia, dysfibrinogenemia or hypodysfibrinogenemia are usually asymptomatic or poorly symptomatic, although this concept has been recently challenged for patients with dysfibrinogenemia and hypodysfibrinogenemia showing a possible more significant thrombotic tendency compared to the risk of bleeding [60,61]. Patients with afibrinogenemia may show severe clinical symptoms since their early infancy; the most common are mucocutaneous bleeding, soft tissue and joint bleeding and prolonged bleeding from the umbilical cord. Women may experience menometrorrhagia and first-trimester abortion is common. Central nervous system (CNS) hemorrhages are not uncommon and post-surgical bleeding are described in 40% of untreated patients. Episodes of thromboembolisms in afibrinogenemia have been reported, often independently from replacement therapy, probably triggered by the excess of unbound circulating thrombin. Different gene mutations may predict bleeding or thrombotic risk in dysfibrinogenemia [61]. Replacement therapy with fibrinogen concentrate represents the first choice for obtaining an effective hemostasis. Conventional treatment is on-demand; to stop bleeding, fibrinogen levels should be raised to at least 100 mg/dL with infusion of 50–100 mg/kg concentrate, followed by doses aimed at maintaining maintaining levels >50 mg/dL, until resolution. These levels are also indicated for haemostatic coverage in surgery [62,63]. Effective long-term secondary prophylaxis with fibrinogen administration every 7 to 14 days has been described after CNS haemorrhages or in patients with recurrent joint bleedings [61]. Prophylactic treatment appears necessary for successful completion of pregnancy: fibrinogen replacement must be begun before five weeks of gestation to prevent abortion and maintained >100 mg/dL throughout the pregnancy; a level >150 mg/dL is recommended for delivery [64].

### 3.4. Prothrombin Deficiency

Prothrombin is a plasma glycoprotein and zymogen of a serine protease requiring vitamin K for normal biosynthesis. Its active form is thrombin, with several pro- and anticoagulant effects. It triggers platelet aggregation and promote coagulation by activating regulatory pathways and generating fibrin monomers by cleavage of a specific peptide bond in fibrinogen α- and β- subunits at N-termini [65]. It is encoded by a gene of 21 kb mapped to chromosome 11. Prothrombin deficiency is usually more a qualitative (dysprothrombinemia) than a quantitative (hypoprothrombinemia) defect. A complete deficiency appears to be incompatible with life, while severe defects (FII plasma levels <5%) is characterized by different symptoms, with recurrent muco-cutaneous and gynaecological bleeding and more rarely haemarthroses and muscle bleeds.

Replacement therapy is needed only in severe deficiency, for stopping bleeding or to ensure adequate prophylaxis in case of surgery. No FII concentrate exists, in fact treatment involves the use of prothrombin complex concentrates (PCCs), that contain other vitamin K-dependent coagulation factors (FIX, FX and not systematically FVII). The recommended treatment is administration of PCC at doses of 20–30 IU/kg, to be repeated as needed to maintain haemostatic levels 20%–30%. In case of major surgery it is recommended to achieve and maintain levels of 30%–40% [62,63].

### 3.5. Factor V Deficiency

FV is an essential non-enzymatic cofactor of prothrombinase complex, which catalyses the conversion of prothrombin into thrombin. Furthermore, it contributes to the down-regulation of FVIII by the protein C/S anticoagulant pathway. Although most FV is circulating into the plasma, approximately 20%–25% is present within platelet α-granules. The protein is encoded by a large gene (25 exons, 80 kb size) located on chromosome 1. Bleeding manifestations are poorly correlated with FV plasma level [66] and consist of bruising, mucosal bleeding and menorrhagia; hemarthrosis and deep hematomas are also described. Replacement therapy of FV can be administered only through FFP, since FV concentrates are not available and FV is not present in cryoprecipitate or PCC. FV levels should be raised to at least 15–20 IU/dL by using FFP; the initial dose should be 15–20 mL/kg followed by smaller amounts, adjusting the dosage on the basis of FV plasma level, PT and APTT. Studies of FV recovery recommend maintaining a level of 20%–25% FV activity in case of severe bleeding or for surgery [62,63]. Development of alloantibodies to FV is a very rare complication of inherited FV deficiency; inhibitors can be neutralized using large amounts of FFP. Platelet concentrates are an alternative source of FV, that can be used in combination with FFP in these situations [67]. A FV concentrate has been developed and a study is in preparation for its orphan drug designation by European Medicine Agency (EMA) and Food and Drug Administration (FDA).

### 3.6. Combined Factor V and Factor VIII Deficiency

The disorder is characterized by concomitantly low levels (between 5% and 20%) of both coagulant activity and antigen [68]. The disease is mainly caused by mutations in the lectin mannose 1 (*LMAN*1) gene, generating a LMAN1 transmembrane protein, which facilitates the intracellular transports of both proteins. Less frequently, mutations in the MCFD2 gene encoding multiple coagulation factor deficiency protein acting as a cofactor for LMAN1 for the recruiting of FV and FVIII within the endoplasmic reticulum are also responsible for the disorder. The combined deficiency does not increase bleeding risk observed in patients with single defect. Symptoms are usually mild with a predominance of mucocutaneous manifestations and postoperative bleeding. Treatment usually relies on on-demand use of FFP (15–20 mL/kg) and DDAVP (0.3 mcg/kg); more severe cases may require FVIII concentrate. Sources of both clotting factors are required always considering their different half-life, which is 36 h for FV and 10–12 h for FVIII [62,63].

### 3.7. Factor VII Deficiency

FVII is a vitamin K-dependent clotting factor with a pivotal role for blood coagulation; in fact, the complex formed between the naturally occurring procoagulant serine protease, activated FVII (FVIIa), and the integral membrane protein tissue factor (TF), exposed on the endothelium upon injury, represents the trigger of blood clotting. The FVIIa-TF complex generates a burst of FIXa and FXa, leading to the formation of a stable fibrin clot [69]. Coagulant and antigenic plasma FVII levels are influenced by genetic and environmental factors, in addition to *F7* polymorphisms. FVII has approximately a 50 KDa weight and circulates in plasma in large amount as inactive form (≈0.5 µg/mL) and in small (50–100 pmoles/L) as active form, which binds to tissue factor. The FVII gene maps to chromosome 13 (13q34) and consists of 9 exons encoding a protein of 406 amino acids. A complete absence of FVII is incompatible with life. FVII deficiency is a heterogeneous hemorrhagic disorder, which ranges in severity from lethal to mild, or even asymptomatic disease [70]. The most frequent symptoms are epistaxis, easy bruising and gum bleeding. In patients affected by severe deficiency CNS and gastrointestinal bleeding, haemarthrosis and muscle hematomas are described. Postoperative bleeding and menorrhagia represent other frequent manifestations [71]. Thrombosis, associated with surgery and replacement therapy or spontaneous, has also been reported in 3%–4% of patients [72]. Different options are available for treatment of FVII deficiency; in recent years, pd-FVII concentrates have been gradually replaced by a recombinant (r-)FVIIa concentrate. Haemostatic levels of FVII amount to 15–20 U/dL and can be obtained and maintained by administering pd-FVII at a dosage of 30–40 IU/kg. Alternatively, rFVIIa can be used at a low dose (15–30 μg/kg body weight (b.w.)) every 4–6 h. Infusions of 25 μg/kg rFVIIa every 3–4 h the first day, followed by bolus at lower doses every 6–8 h are effective for treatment and prevention of postoperative bleeding [62,63]. The short half-life of FVII (3–4 h) requires frequent infusions. Theoretically, this should prevent an effective use of FVII in secondary prophylaxis regimens. However, there is evidence of effective prophylaxis in pediatric patients with CNS bleeding or recurrent haemarthrosis, treated with 30–40 IU/kg pd-FVII or 15–30 μg/kg rFVIIa two or three times per week [73].

### 3.8. Factor X Deficiency

FX is a vitamin K-dependent proteases synthesized by the liver and secreted into the plasma as a zymogen composed of a light and a heavy chain [74], with a concentration of around 10 μg/mL. It is activated by FIXa or by FVIIa and its role is pivotal in the coagulation cascade: FIXa or FVIIa activate FX by cleavage of the Arg194-Ile195 peptide bond located in the heavy chain [75]. The gene coding for FX maps to the long arm of chromosome 13 (13q34), adjacent to FVII gene, and consists of 8 exons over a 27 kb of genomic sequence. FXa is the major physiologic activator of prothrombin.

Inherited FX deficiency is one of the rarest RBD and the variable severity of symptoms correlates poorly with laboratory phenotype, unless severe quantitative deficiency (FX < 1%) is observed. However bleeding from umbilical stump and recurrent hemarthroses may occur in the more severely affected (<1% activity). The administration of PCC at doses of 20–30 IU/kg represents the recommended treatment, to be repeated as needed to maintain haemostatic levels 15%–20%. In case of major surgery it is recommended to attain and maintain levels of 30%–40% [62,63]. In Germany a freeze-dried human high content coagulation FX/FIX has been developed. It was used at dosage of 10–20 IU/kg, in prophylaxis regimen two or three times per week in patients with FX < 1% and more than 20 hematomas or haemarthroses per year. Efficacy and safety results are encouraging [76]. Recently, a new FX concentrate has been produced by BPL (Elstree, UK).

### 3.9. Factor XI Deficiency

FXI is a glycoprotein that consists of two identical polypeptide subunits, circulating into the plasma as non-covalent complex with high-molecular-weight kininogen [77]. The physiologic activator of FXI is thrombin, that converts zymogen FXI to the serine protease FXIa. FXIa is not only able to activate FIX, promoting early step coagulation cascade, but also to diminishes fibrinolysis, activating thrombin-activatable fibrinolysis inhibitor (TAFI). The human FXI gene (*F11*) comprises 15 exons, and spans about 23 kb on the long arm of chromosome 4 (4q35.2). The highest prevalence of FXI deficiency has been observed in Ashkenazi Jews, with a heterozygosity of 9%. Homozygotes or compound heterozygotes have a FXI activity <15 U/dL, while heterozygotes have an activity range of 25–70 U/dL. Bleeding tendency does not correlate with plasma FXI levels. Spontaneous bleeding is rare in patients with severe FXI deficiency. The most frequent manifestations are oral, post-traumatic and post-operative bleeding [78], menorrhagia is common in affected women and postpartum haemorrhage occurs in 20%–30% of affected women.

Treatment is based on antifibrinolytic agents, fresh frozen plasma (FFP) and pd-FXI concentrate. Replacement therapy is usually represented by FFP, because some cases of potentially life-threatening thromboembolic complications have been observed when using pd-FXI concentrate in patients with cardiovascular risk factors and post-infusion levels greater than 100%. Thus, pd-FXI concentrate should be avoided in elderly and in subjects with pre-existing cardiovascular disease [79]. Notwithstanding these side effects, pd-FXI have been successfully used in many patients. Different studies have shown a 90% recovery of FXI after infusion and a half-life of 46–52 h. Doses greater than 30 IU/kg b.w. must be avoided, anyway, 15–20 IU/kg b.w. are sufficient to maintain a peak between 50 and 70 IU/dL [80,81]. Replacement therapy is indicated in patients with FXI levels <5 UI/dL, history of post-traumatic or postsurgical bleeding. Inhibitory antibodies can develop in patients treated with FFP; this complication seems to be particularly associated to homozygosity for stop mutations Glu117. A careful assessment of risk and benefit must be done before treating with FFP or pd-FXI patients with FXI deficiency. In surgery and in patients with inhibitors rFVIIa has been successfully used.

### 3.10. Factor XIII Deficiency

The transglutaminase enzyme FXIII plays a pivotal role in hemostasis by catalysing the cross-linking of fibrin and enhancing fibrin interaction with different platelet integrins and matrix protein throughout thrombus formation. These processes are responsible for strengthening and stabilizing the clot FXIII circulates in plasma as a tetramer (A_2_B_2_) composed of 2 catalytic A subunits (FXIII-A) and 2 carrier B subunits (FXIII-B) [82]. FXIII-A is produced by cells of bone marrow origin and can be found in platelets, monocytes, macrophage and placenta, as homodimer or linked to circulating platelets into the blood. FXIII-B is synthesized in the liver and can be found in plasma as free form or bound to FXIII-A in heterotetrameric form, acting as protection against FXIII-A proteolytic degradation and inactivation. *F13A* gene maps to chromosome 6 (p24-25) and spans over 160 Kb. It consists of 15 exons encoding for a mature protein of 731 amino acids. The F13B gene is located on the long arm of chromosome 1 (q32-32.1) and contains 12 exons encoding for a mature protein of 641 amino acids.

Inherited FXIII deficiency is mostly caused by mutations in *F13A* resulting in a quantitative and/or qualitative FXIII-A deficiency with a usually severe bleeding tendency. FXIII subunit B deficiency is very rare and only a few families have been reported, with milder bleeding tendency. Umbilical cord and CNS bleeding are reported in 80% and 30%, respectively [83]. Other clinical manifestations include severe bleeding in joints or soft tissue, poor wound healing and spontaneous abortions. Delayed post-operative bleeding is often observed because of normal clot formation, but early fibrinolysis. Patients with homozygous FXIII deficiency present with severe bleeding symptoms and plasma levels <1%, whereas those with heterozygous mutations have rarely mild symptoms. Once the diagnosis is confirmed, prophylactic therapy with FXIII replacement is fundamental to prevent severe disability or death due to spontaneous bleeding episodes. Long-term prophylaxis is feasible, as 2% to 5% FXIII plasma levels are sufficient to prevent severe bleeding. FXIII in vivo half-life is 11 to 14 days, prompting replacement every 3–4 weeks. Prophylactic treatment is recommended in FXIII-A deficient women during pregnancy to prevent abortion [84]. In addition to the safe and effective pd-XIII, a new recombinant concentrate is available. r-FXIII consists of FXIII-A_2_ dimer, which can bind to circulating FXIII-B subunits, producing the tetrameric molecule. EMA has approved the drug for patients with more 6 years and in Italy it can be used only for prophylactic treatment. Administration of FXIII at dosage of 20–40 IU/kg is able to maintain levels >3%, which are efficacious for treatment of acute bleeding, prevention of post-operative bleeding and as a prophylaxis [62,63]. A similar dose (35 U/kg) is usually recommended also with rFXIII [53].

## 4. Vitamin K-dependent coagulation factors deficiency

The deficiency affects all vitamin K dependent factors: FII, FVII, FIX, FX, C and S protein. VKCFD is due to mutations in genes encoding necessary enzymes for post-translational change and vitamin K metabolism (GGCX located on chromosome 2 and VKOR, located on chromosome 16). Plasma levels of each factor may range between 1% and 30%, VKCD usually presents early in life with CNS or umbilical cord bleeding. Children with severe disease may present with skeletal abnormalities and mild conductive hearing loss. Older patients can present with mucocutaneous or postsurgical bleeding. Treatment with oral or parenteral vitamin K1 should be promptly started. When response is inadequate, replacement therapy is needed in acute bleeding or prior to surgery, using prothrombin complex concentrates (PCCs) or FFP to obtain circulating levels of each factor at least 15%–20% [85].

## 5. Conclusions

Inherited bleeding disorders are extremely heterogeneous, ranging from the most frequent VWD to very rare severe prothrombin or FXIII deficiencies, with different bleeding tendency. The residual coagulant activity is not always strictly associated to bleeding manifestation severity and molecular diagnosis may help in understanding better the bleeding phenotype. The rarity of these disorders justifies the joint efforts of sharing clinical data through appropriate registries to better understand the outcome in different countries. This would in turn result in a better dissemination of clinical and laboratory data with advices for the best treatment options available. Different treatment options, including plasma-derived virus-inactivated concentrates or some recombinant concentrates are available to improve the long-term outcomes. Treatment on-demand for bleeding or surgery, periodic or long-term prophylaxis in selected disorders and/or conditions, as pregnancy, enables to achieve optimal management of patients with VWD or RBDs. Unfortunately, in developing countries both diagnostic and therapeutic opportunities are still limited and future efforts should be aimed at reducing these inequalities.

## Figures and Tables

**Table 1:** Classification of von Willebrand disease, modified from Sadler et al. 2006 [15].

**Types**	**Quantitative Deficiency of VWF**
**Type 1**	Partial quantitative deficiency of VWF (~60%–70% of all cases)
**Type 3**	Virtually complete deficiency of VWF (~1%–2% of all cases)
	**Qualitative Deficiency of VWF**
**Type 2**	Qualitative deficiency of VWF (~25%–30% of cases)
**Type 2A**	Qualitative variants with decreased platelet-dependent function associated with the absence of high and intermediate-molecular-weight VWF multimers
**Type 2B**	Qualitative variants with increased affinity for platelet GpIb
**Type 2M**	Qualitative variants with decreased platelet-dependent function not caused by the absence of high-molecular-weight VWF multimers
**Type 2N**	Qualitative variants with markedly decreased affinity for FVIII

**Table 2:** Laboratory patterns in von Willebrand disease [14].

Laboratory Assay	Pathophysiological Significance	Type 1 VWD	Type 2A VWD	Type 2B VWD	Type 2M VWD	Type 2N VWD	Type 3 VWD
APTT	Reflects the degree of reduction of FVIII	Prolonged or normal	Prolonged or normal	Prolonged or normal	Prolonged or normal	Prolonged	Prolonged
Platelet count	Increased affinity for GpIb in type 2B	Normal	Normal	Low or normal	Normal	Normal	Normal
PFA-100 (CT)	Simulates primary hemostasis after injury to a small vessel	Prolonged or normal	Prolonged, no closure	Prolonged, no closure	Prolonged, no closure	Normal	Prolonged, no closure
FVIII:C	FVIII-VWF interaction	Low or normal	Low or normal	Low or normal	Low or normal	Proportionally low	Low (<10 IU/dL)
VWF:AG	Antigen concentration	Low	Low or normal	Low or normal	Normal or low	Normal or low	Very low (<5 IU/dL)
VWF:RCo	VWF–GPIb interaction as mediated by ristocetin in vitro	Low	Very Low (<20 IU/dL)	Variably low	Low	Normal or low	Very low (<5 IU/dL)
VWF:CB	VWF–collagen interaction	Low, rarely normal	Very low	Low	Low or normal	Normal or low	Very low (<5 IU/dL)
VWF:RCo/VWF:Ag ratio		Normal (>0.6)	Low (<0.6)	Low (<0.6)	Low or normal	Normal (>0.6)	Variable
RIPA using patient’platelets	Threshold ristocetin concentration inducing patient’s platelet-rich plasma aggregation	Reduced or normal	Reduced or normal	Occurs at lower concentrations than in normal subjects	Reduced or normal	Normal	Absent
VWF multimer pattern	Multimeric composition of VWF	Normal pattern, VWF reduced	Large to intermediate multimers missing	Large multimers missing	Normal VWF multimer distribution (but with possible abnormal bands)	Normal	Multimers absent

APTT, activated partial thromboplastin time; FVIII:C, factor VIII coagulant; VWF:AG, VWF antigen; VWF:RCO, VWF ristocetin cofactor; VWF:CB, VWF collagen binding; RIPA, ristocetin-induced platelet agglutination; CT, closure time; VWD, von Willebrand Disease.

**Table 3:** VWF/FVIII concentrates licensed in Europe.

Product	Brand	Purification	Viral Inactivation	VWF:RCo/Ag ° (Ratio)	VWF:RCo/FVIII ° (Ratio)
Alphanate	Grifols	Heparin ligand chromatography	S/D + dry heat (80°, 72 h)	0.47 ± 0.1	0.91 ± 0.2
Fanhdi	Grifols	Heparin ligand chromatography	S/D + dry heat (80°, 72 h)	0.47 ± 0.1	1.04 ± 0.1
Haemate P/Humate P	CSL Behring	Multiple precipitation	Pasteurization (60°, 10 h)	0.59 ± 0.1	2.45 ± 0.3
Immunate	Baxter	Ion exchange chromatography	S/D + vapor heat (60°, 10 h)	0.47	1.1
Wilate	Octapharma	Ion exchange + size exclusion chromatography	S/D + dry heat (100°, 2 h)	-	0.9
Wilfactin	LFB	Ion exchange + affinity	S/D, 35 nm filtration, dry heat (80°, 72 h)	0.95	50

Note: S/D: solvent/detergent; ° Data from Reference [14].

**Table 4:** Indications for replacement therapy in VWD Reference [14].

**Indications for Replacement Therapy in VWD Reference [14]**
**Spontaneous or post-traumatic severe bleeding** Single or daily doses of 50 IU/kg of VWF to maintain FVIII:C levels >50 U/dL until bleeding stops (usually 7–10 days) * **Spontaneous or post-traumatic mild to moderate bleeding** Single or daily doses of 20–40 IU/kg of VWF to maintain FVIII:C levels >30 U/dL until bleeding stops (usually 1–3 days) *
**Major surgery** Daily doses of 50–60 IU/kg of VWF to maintain pre-operative FVIII:C and VWF:RCo levels of 80–100 U/dL until 36 h postoperatively and then >50 U/dL until healing is complete (usually 7–10 days) * Measure plasma levels of FVIII:C (and VWF:RCo ) every 12 h on the day of surgery, then ever 24 h Usual thrombo-prophylactic treatment with LMWH should be implemented in patients at high risk of venous thrombosis **Minor surgery** Daily or every other day doses of 30–60 IU/kg of VWF to maintain FVIII:C levels >30 U/dL until healing is complete (usually 1–5 days) * **Dental extractions** Single dose of 20–40 IU/kg of VWF to maintain FVIII:C levels >50 U/dL for 12 h * **Delivery and puerperium** Daily doses of 50 IU/kg of VWF to maintain FVIII:C levels >50 U/dL for 3–4 days
**In type 3 patients with alloantibodies** all plasma concentrates containing VWF must be avoided because of the risk of anaphylactic reactions. r-FVII (Recombinant FVIII) administered at very high doses by continuous intravenous infusion, or r-FVIIa can be used instead

Note: Dosing should be based on VWF:RCo content where this is available. * These dosages are indicated for VWD patients with FVIII:C/VWF:RCo levels <10 U/dL.

**Table 5:** Clinical symptoms, laboratory and molecular diagnosis in Rare Bleeding Disorders (RBDs).

Deficiency	Plasma Level (µg/mL)	Bleeding Symptoms	Laboratory Diagnosis	Prevalence	Gene (Chromosome)
Fibrinogen	1500–4000	Umbilical cord, haemarthrosis, mucosal tract, menorrhagia, first trimester abortion, CNS Venous and arterial thromboembolism are reported	*Afibrinogenemia:* APTT↑↑, PT↑↑, TT↑↑ *Dys-Hypofibrinogenemia:* APTT↑, PT↑↑, TT↑	1:1,000,000	*FGA*, *FGB*, *FGG* (4q28)
*F II*	100	Umbilical cord, haemarthrosis, mucosal tract	APTT↑, PT↑, TT normal	1:2,000,000	F2 (11p11-q12)
*F V*	10	Mucosal tract, postoperative	APTT↑, PT↑, TT normal	1:1,000,000	F5 (1q24.2)
*F VII*	0.13–1.0	Mucosal tract, haemarthrosis, haematomas, neonatal CNS haemorrhage	APTT normal, PT↑, TT normal	1:500,000	F7 (13q34)
*F X*	10	Umbilical cord, haemarthrosis, haematomas, CNS haemorrhages	APTT↑, PT↑, TT normal	1:1,000,000	F10 (13q34)
*F XI*	3–6	Oral cavity, post-traumatic, postoperative	APTT↑, PT normal, TT normal	1:1,000,000	F11 (4q35.2)
*F XIII*	10–20	Umbilical cord, spontaneous CNS haemorrhages, miscarriages, abnormal scarring	APTT normal, PT normal, TT normal Specific FXIII assay	1:2000000	F13A1 (6p24-p25) F13B (1q31-q32.1)
*FV + FVIII*	As for each factor	Mucosal tract, postoperative	APTT↑, PT↑, TT normal	1:1,000,000	LMAN1 (18q21.3-q22) MCFD2 (2p21-p16.3)
*VKCFD*	As for each factor	Umbilical cord, CNS haemorrhages, postoperative *Children may show skeletal abnormalities*	APTT↑, PT↑↑, TT normal	<50 families	GGCX (2p12) VKORC1 (16p11.2)

Note: CNS, central nervous system; APTT, activated partial thromboplastin time; PT, prothrombin time.

**Table 6:** Replacement therapy for RBDs.

Deficient Factor	Plasma Half-Life	Haemostatic Level (U/mL)	Available Clotting Factor Concentrate	On-Demand Dosages	Long-Term Prophylaxis Dosages	Frequency of Prophylaxis
Fibrinogen	2–4 days	50 (mg/dL)	pd-fibrinogen	50–100 mg/kg	20–30 mg/kg	Once–Twice a week
*F II*	3–4 days	20–30	pd-PCC	20–30 IU/kg	20–30 IU/kg	Once a week
*F V*	36 h	15–20	No concentrate	-	Not indicated	-
*F VII*	3–4 h	15–20	pd-FVII rFVIIa	30–40 IU/kg 15–30 mcg/kg	30–40 IU/kg 20–40 mcg/kg	Thrice weekly Twice weekly
*F X*	40–60 h	15–20	pd-PCC FX/FIX concentrate FX concentrate	20–30 IU/kg	20–40 IU/kg	Twice weekly
*F XI*	40–70 h	15–20	pd-FXI	15–20 IU/kg	Not indicated	-
*F XIII*	11–14 days	2–5	pd-FXIII r-FXIII	20–40 IU/kg 35 IU/kg	20–40 IU/kg 35 IU/kg	Every 3–4 weeks
*FV + FVIII*	36 h (FV) 10–12 h (FVIII)	15–20	Only pd-FVIII or r-FVIII	FVIII: 25–40 IU/kg Plasma 15–25 mL/kg	Not indicated	-
*VKCFD*	As for each factor	15–20	pd-PCC	20–30 IU/kg	No data	-

Note: pd, plasma-derived; r, recombinant; PCC, prothrombin complex concentrate.
